# Estimation of Traffic Stream Density Using Connected Vehicle Data: Linear and Nonlinear Filtering Approaches

**DOI:** 10.3390/s20154066

**Published:** 2020-07-22

**Authors:** Mohammad A. Aljamal, Hossam M. Abdelghaffar, Hesham A. Rakha

**Affiliations:** 1 Charles E. Via, Jr. Department of Civil and Environmental Engineering, Center for Sustainable Mobility, Virginia Tech Transportation Institute, Virginia Tech, Blacksburg, VA 24061, USA; m7md92@vt.edu; 2 Department of Computer Engineering and Systems, Engineering Faculty, Mansoura University, Mansoura 35516, Egypt; hossam_wahed@mans.edu.eg or; 3 Center for Sustainable Mobility, Virginia Tech Transportation Institute, Virginia Tech, Blacksburg, VA 24061, USA

**Keywords:** traffic density, connected vehicles, real-time estimation, particle filter, Kalman filter

## Abstract

The paper presents a nonlinear filtering approach to estimate the traffic stream density on signalized approaches based solely on connected vehicle (CV) data. Specifically, a particle filter (PF) is developed to produce reliable traffic density estimates using CV travel-time measurements. Traffic flow continuity is used to derive the state equation, whereas the measurement equation is derived from the hydrodynamic traffic flow relationship. Subsequently, the PF filtering approach is compared to linear estimation approaches; namely, a Kalman filter (KF) and an adaptive KF (AKF). Simulated data are used to evaluate the performance of the three estimation techniques on a signalized approach experiencing oversaturated conditions. Results demonstrate that the three techniques produce accurate estimates—with the KF, surprisingly, being the most accurate of the three techniques. A sensitivity of the estimation techniques to various factors including the CV level of market penetration, the initial conditions, and the number of particles in the PF is also presented. As expected, the study demonstrates that the accuracy of the PF estimation increases as the number of particles increases. Furthermore, the accuracy of the density estimate increases as the level of CV market penetration increases. The results indicate that the KF is least sensitive to the initial vehicle count estimate, while the PF is most sensitive to the initial condition. In conclusion, the study demonstrates that a simple linear estimation approach is best suited for the proposed application.

## 1. Introduction

Real-time traffic estimation has received increased attention with the introduction of advanced applications and technologies such as intelligent transportation systems (ITSs). Adaptive traffic signal controllers require real-time traffic state estimation to improve intersection performance, as real-time estimation plays a major role in capturing variations in traffic behavior (e.g., nonrecurrent changes). As inputs to traffic signal controllers, traffic state variables (e.g., travel time and traffic density) assist with green time allocation and help to enhance intersection performance by reducing traffic delays, vehicle emissions, and fuel consumption [1,2]. Several estimation techniques have been developed to estimate traffic state variables [3,4,5,6,7]. In some previous studies, the traffic state system has been treated as a linear system [4,7,8,9,10]. Other studies have considered the system as nonlinear [3,11,12,13]. For linear system models, a Kalman filter (KF) has been widely deployed to produce accurate estimates [4,7,10,14] due to its simplicity and applicability in the field. The KF assumes linear system transitions with a Gaussian distribution for the probability density function (PDF) of the system and measurement noise. For nonlinear system models, an extended KF (EKF) has been utilized in estimation [11,15,16]. The EKF also assumes that the PDF distribution is Gaussian. The EKF is derived by linearizing the system using a Taylor series expansion by calculating the Jacobian expression. However, it was found that use of the EKF approach is only valid if the system is near linearity during the updating time [17], and thus, large errors may result from linearization. In addition, the task of deriving the Jacobian matrices may cause implementation difficulties [18]. A more robust nonlinear approach is a particle filter (PF), which has been frequently employed in the literature to handle nonlinear dynamic problems [12,19,20]. The PF approach is a Monte Carlo sequential solution that deals with nonlinear system transitions without the assumption of the PDF noise distribution [21,22]. In this paper, a PF approach is developed to estimate the traffic stream density along signalized intersection approaches using only connected vehicle (CV) data. Moreover, the paper compares the performance of the PF to the KF and adaptive KF (AKF) approaches.

Traffic density is defined as the number of vehicles per unit length on a specific roadway segment [23]. Estimating the traffic stream density is critical in the development of effective traffic controllers [24]. For instance in the case of freeways, identifying bottleneck locations in the early stages is critical in developing congestion mitigation strategies that include ramp metering, variable speed limits, and traffic routing. For signalized segments, the traffic density measures are crucial for either traffic signal performance [25,26,27] or traffic signal optimization [28,29,30]. Hence, traffic density measures must be precisely estimated to represent traffic demands at each signalized intersection approach. Once accurate measurements are obtained, efficient adaptive traffic signal controllers can be developed. However, determining traffic density is not a trivial task and cannot be directly measured in the field since it is a spatial measurement. Consequently, traffic stream density is typically based on estimations.

Previous research has utilized different data sources, such as stationary sensors (e.g., loop detectors), fused data (combining two distinct data sources), and CV data to estimate traffic stream density. Traffic density estimates can be measured using video detection systems, but this is difficult due to the high cost of the infrastructure and the limited visibility of roadway segments [4]. Time-occupancy measurements from loop detectors are used as an alternative data source to estimate the traffic density [31]. However, time-occupancy measurements only represent the temporal density estimates around the location of the detector. A recent study introduced a relationship between time-occupancy and space-occupancy to estimate traffic density by dividing the link into small segments and installing detectors on all of the small segments [32], but the installation cost is high. A more common way of estimating the traffic density is the use of the traffic flow continuity equation (input–output approach), which considers two traffic counting stations, one at the entrance and the other at the end of the link [33]. Vigos et al. [4] proposed a robust linear KF approach with at least three loop detectors to estimate the traffic density along signalized approaches. However, the implementation cost is high. Another study [34] employed two conventional loop detectors to estimate the traffic density using the flow continuity equation. The two loop detectors provide the estimation model with traffic flow and occupancy data. Bhouri et al. [35] proposed a KF approach to estimate the traffic stream density along a freeway segment using both loop detectors and a recorded film [35]. One commonality about the use of fixed sensors is that they are subject to detection failures and thus always produce errors in their data [36,37].

Recent research has fused different data sources to estimate the traffic stream density along certain roadway sections, increasing the accuracy of the estimate over using just one data source [7,14]. Many works have employed the KF approach [14,38,39]. For instance, traffic flow data at the entrance and the exit of the roadway section observed from stationary sensors together with CV data were used to estimate the traffic density [38]. The CV data provided travel-time measurements to correct the prior estimate from the state equation. Another study has utilized fused loop and CV measurements to estimate the traffic density in a freeway section [19]. In that study, the authors derived the estimation model using the PF estimation approach, considering two sources of measurements: (1) loop detectors, and (2) fusing loop detectors and CVs. They obtained a 30% reduction in the mean absolute percentage error from the fused measurements compared to the measurements from loop detectors, demonstrating that more data sources produce more-accurate outcomes. However, the use of different data sources requires more computational cost in both time and memory as the data include both trivial (data that are not needed) and nontrivial (data that are needed) information.

Limited studies have used CV data as the only source of inputs to estimate the traffic stream density [7,9,10]. These studies developed the linear KF estimator approach. The CV data used were the number of CVs at the entry and at the exit of the tested roadway section, in addition to the travel time experienced for the CVs to traverse the tested section. Moreover, Aljamal et al. [7] demonstrated that treating the estimation interval time as a variable instead of a fixed value is mandatory when dealing with only CV data, as the variable approach always ensures that sufficient information is gathered from the CVs in every estimation interval. This approach enhances the accuracy of the estimation, especially for the scenarios with low CV level of market penetration (LMP) rate. The estimation time interval for this study is therefore defined as the time when an exact number of CVs (i.e., 5 vehicles) reach the end of the tested link.

Several researchers have employed the PF approach to improve traffic stream estimates for different transportation applications, including traffic flow [12,13], travel time [3,20], and traffic speed [40]. In one study, magnetic loop detectors were placed at the boundaries of the tested freeway section to estimate the traffic flow, and a PF estimation approach was developed using traffic flow and speed measurements [12]. In another study, Mihaylova et al. [13] developed two nonlinear approaches, an unscented KF and a PF, to produce real-time traffic flow estimates in a freeway network using data from stationary sensors. They found that the PF approach outperformed the unscented KF. Chen et al. [20] proposed a time series speed equation to estimate traffic speed. They claimed that the traffic system is nonlinear and thus presented two nonlinear approaches, a PF and an ensemble KF, using available speed measurements from loop detectors. They found that the PF approach is more accurate than the ensemble KF. Another study developed a PF estimation approach for travel-time predictions using real-time and historical data [3]. They used the historical data to generate particles as opposed to using a state-transition model. In addition, a comparison between the PF, KF, and k-nearest neighbor estimators found that the PF is the most accurate approach. CV data were employed to estimate the traffic speed and flow using the PF approach [40]. In that study, each link in the network was assumed to have base stations to retrieve and transfer the data. Results found that other data sources (e.g., loop detectors) should be incorporated with CVs to enhance the estimation performance. However, our recent study developed a KF approach, showing that the use of CV data alone is sufficient to obtain accurate results [7].

In summary, the existing literature shows that the PF has been widely used to address nonlinear systems and has been proven to outperform other nonlinear estimation techniques; however, to our best of knowledge in the application of traffic stream density estimation, only a few studies have applied the PF approach using data from stationary sensors and fusing data from different sources. In addition, no comparison between the PF and the linear KF has been reported. Therefore, the PF was adopted in this study. The primary objective of this study is to develop a nonlinear PF estimation approach to estimate the traffic stream density based solely on CV data on signalized approaches. Subsequently, we compare the PF approach to linear estimation approaches—namely, KF and AKF—to identify the best approach for the application of the traffic density estimation, given that no comparison has been reported in the literature between these filtering techniques. Consequently, this research will recommend a specific approach to estimate the traffic stream density. The proposed three approaches are employed to estimate the vehicle counts based solely on CV data. In addition, this study also investigates the sensitivity of the proposed estimation approaches to several factors, such as the LMP rate of the CVs, the initial conditions, and the number of PF particles.

The paper is organized as follows: Section 2 describes the problem formulation and the estimation approaches. Section 3 discusses the findings from applying the estimation approaches. Section 4 includes the conclusions of the study and the proposed future work.

## 2. Problem Formulation and Estimation Approaches

First, Section 2.1 formulates the research problem using a state-space model. Then, three different estimation approaches are described: the PF (Section 2.2.1), the KF (Section 2.2.2) and the AKF (Section 2.2.3).

### 2.1. State-Space Model

**Figure 1:**
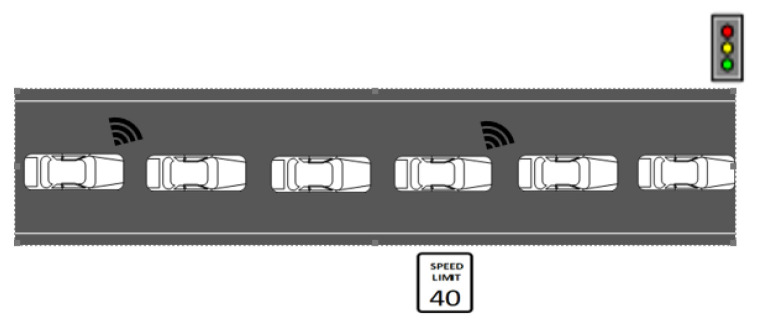
Tested link section includes connected vehicles (CVs) and non-CVs.

The state-space model is represented by a state equation and a measurement equation. The state equation describes how the system behaves and provides a prior knowledge of the estimation. The measurement equation is used to help correct and improve the prior estimation. In this study, the goal is to estimate the number of vehicles on signalized links using only CV data, as depicted in Figure 1, where CVs are the vehicles that have the connection icon (e.g., the first vehicle on the left). The only information that is needed in practice is as follows: (1) the traffic flow of CVs observed at the tested link’s entrance and exit. (2) the travel time of each CV. Vehicle-to-Infrastructure (V2I) communication can provide this information to the traffic signal controller.

The model is formulated using the derived state-space equations in [7]. The state equation, Equation (1), is based on the continuity equation of traffic flow; whereas the measurement equation, Equation (2), is based on the traffic flow hydrodynamic relationship, based on measurements of the average travel time of CVs. In Equation (1), the number of vehicles is computed by continuously adding the difference of the number of vehicles that enter and exit the tested section to the cumulative number of vehicles traveling along the section previously computed.

(1) N(t)=N(t−Δt)+u(t)

(2) $$TT\left(t\right)=H\left(t\right)\times N\left(t\right)$$

where N(t) is the number of vehicles traversing the link at time *t*, N(t−Δt) is the number of vehicles traversing the link in the preceding time interval, and u(t) is the system inputs, as described in Equation (3).

(3) $$u\left(t\right)=\frac{\Delta t\;[q^{in}\left(t\right)-q^{out}\left(t\right)]}{\max(\rho_{actual},\rho_{min})}$$

where $q^{in}$ and $q^{out}$ represent the flow of CVs entering and exiting the link, respectively, during Δt. ρ is the CVs’ LMP, defined as the ratio of CV count to total vehicle count. In the state equation, the ρ variable is set to be the maximum number of the actual ρ ($\rho_{actual}$) and a predefined minimum value of ρ ($\rho_{min}$). $\rho_{actual}$ can be obtained from historical data. $\rho_{min}$ is introduced to avoid producing large errors in the state equation since a single ρ value is used to approximate the two ρ values (upstream and downstream of the tested link) [7]. In this study, $\rho_{min}$ is set to be equal to 0.5; more details about the system state representation can be found in [7]. It should be noted that the ρ variable is the main noise source in the system, and thus, there is an urgent need to develop the measurement equation to fix these errors. In Equation (2), TT is the average vehicle travel time, H(t) is a vector that transforms the vehicle counts to travel times. H(t) is derived from the hydrodynamic relationship between the macroscopic traffic parameters (flow, density, and space-mean speed), as presented in Equation (4).

(4) $$H\left(t\right)=\frac{1}{\bar{q}\left(t\right)}=\frac{2\;\times \rho_{actual}}{q^{in}\left(t\right)+q^{out}\left(t\right)}$$

### 2.2. Estimation Approaches

As mentioned earlier, the KF and the AKF are considered linear estimators that can efficiently handle linear state-space systems. However, in the proposed state-space equations, we suspect some nonlinearity coming from the ρ variable, which raises the question, would a nonlinear filter improve the estimation performance? For this purpose, this study develops a nonlinear PF approach to estimate the vehicle counts along the signalized link. This section presents the formulation of the three approaches used to estimate the vehicle counts using only CV data along signalized approaches. The three techniques are the proposed PF, the KF [7], and the AKF [10].

#### 2.2.1. The PF Approach

The PF approach is used to solve nonlinear state-space systems with no form restrictions on the initial state and noise distributions. For instance, the PF can deal with any arbitrary PDF distribution [21]. The PF approach is used to estimate the posterior PDF of the state vehicle count variable (*N*) given some measurements of CV travel times (TT) by assigning *k* number of particles (samples). Each particle has a certain relative weight (*w*). When a new measurement is received, the particles’ locations and weights are updated. It should be noted that the particles with low relative weight values are replaced with new particles (resampling) so that the system keeps only the important particles. The estimates are then calculated using the average value of the remaining particles. The following steps are used to implement the proposed PF approach:

1. Initialization: t=0; where *t* is the time interval.
  (a) ${\hat{N}}^{+}\left(0\right)$, *R*, *V*, and *k*,
    where ${\hat{N}}^{+}\left(0\right)$ is the initial vehicle count estimate; *R* is the measurement’s covariance error; and *V* is the variance of the initial vehicle count estimate, which is used to randomly generate the initial particles’ locations around ${\hat{N}}^{+}\left(0\right)$.
  (b) Generate *k* particles’ locations randomly, from 1 to *K*, from the initial prior Gaussian distribution $P(N_{0})$.
    (5) $$N^{k}\left(0\right)∼P\left(N_{0}\right)$$
2. For *t* = 1:T.
  (a) Update the locations ($N^{k}\left(t\right)$), measurements ($TT^{k}\left(t\right)$), and weights ($w^{k}\left(t\right)$) of the particles.
    (6) $$N^{k}\left(t\right)=N^{k}\left(t-\Delta t\right)+u\left(t\right)$$
    (7) $$TT^{k}\left(t\right)=\;H\left(t\right)\times N^{k}\left(t\right)$$
    (8) $$w^{k}\left(t\right)=\frac{1}{\sqrt{2\pi R}}\;e^{-{\left(TT-TT^{k}\left(t\right)\right)}^{2}/2R}$$
    where TT is the observed measurement from the CVs. The weights are then normalized using the following equation, ${\hat{w}}^{k}\left(t\right)=w^{k}\left(t\right)/\sum_{k=1}^{K}w^{k}\left(t\right)$.
  (b) Replace the low-weighted particles with new particles (resampling [21]). After a few iterations in the PF process, the weight will focus on a few particles only and most particles will have insignificant weights, resulting in sample degeneracy [41]. The resampling process is therefore used to tackle the degeneracy problem. It should be noted that the highly weighted particles are used to compute the PF posterior estimate.
  (c) Compute the PF posterior estimate: The PF posterior estimate is computed as the average value of the remaining particles (particles with high weights), as shown in Equation (9).
    (9) $${\hat{N}}^{+}\left(t\right)=\frac{1}{K}\sum_{k=1}^{K}N^{k}\left(t\right)$$
  (d) Next time step (t+Δt): When 5 new CVs traverse the link, return to step 2a.

#### 2.2.2. The KF Approach

The KF approach is a linear quadratic estimator. It has been proven to be the best for estimating linear systems with Gaussian noise [42]. The KF estimation approach can be solved using the following steps:

1. Initialization: t=0; where *t* is the time interval.
  (a) ${\hat{N}}^{+}\left(0\right)$, *R*, and ${\hat{P}}^{+}\left(0\right)$,
    where ${\hat{P}}^{+}\left(0\right)$ is the initial posterior error covariance estimate for the state system.
2. For *t* = 1:T.
  (a) Prior estimates:
    (10) $${\hat{N}}^{-}\left(t\right)={\hat{N}}^{+}\left(t-\Delta t\right)+u\left(t\right)$$
    (11) $$\hat{TT}\left(t\right)=\;H\;\left(t\right)\;\times \;{\hat{N}}^{-}\left(t\right)$$
    (12) $${\hat{P}}^{-}\left(t\right)=\;{\hat{P}}^{+}\left(t-\Delta t\right)$$
    where ${\hat{N}}^{-}$ is an estimate of a priori vehicle count, $\hat{TT}$ is the estimated average travel time, and ${\hat{P}}^{-}$ is the a priori covariance estimate for the state system.
  (b) Correction: The correction uses the prior estimate and the new measurement (i.e., the CV average travel time) to compute the Kalman gain (*G*).
    (13) $$G\left(t\right)=\;{\hat{P}}^{-}\left(t\right)H{\left(t\right)}^{T}\;{\left[H\left(t\right){\hat{P}}^{-}\left(t\right)\;H{\left(t\right)}^{T}+R\right]}^{-1}$$
  (c) Posterior state estimates:
    (14) $${\hat{N}}^{+}\left(t\right)={\hat{N}}^{-}\left(t\right)+G\left(t\right)\;\left[TT\left(t\right)-\;\hat{TT\;}\left(t\right)\right]$$
    (15) $${\hat{P}}^{+}\left(t\right)={\hat{P}}^{-}\left(t\right)\times \left[1-H\;\left(t\right)\;G\;\left(t\right)\right]$$
    where ${\hat{N}}^{+}$ is the posterior vehicle count estimate, and ${\hat{P}}^{+}$ is the posterior error covariance estimate.
  (d) Next time step (t+Δt): When 5 new CVs traverse the link, return to step 2a.

#### 2.2.3. The AKF Approach

The AKF approach is presented to estimate the total number of vehicles, using real-time noise error estimates in the state and measurement systems (i.e., mean and variance values). It should be noted that the KF and the AKF approaches use the same equations, but the AKF approach dynamically estimates the noise statistical parameters every estimation step. The vehicle count estimates can be obtained using the following steps:

1. Initialization: t=0; where *t* is the time interval.
  (a) ${\hat{N}}^{+}\left(0\right)$, m(0), and ${\hat{P}}^{+}\left(0\right)$,
    where m(0) is the mean of the noise for the state system.
2. For *t* = 1:T
  (a) Prior estimates:
    (16) $${\hat{N}}^{-}\left(t\right)={\hat{N}}^{+}\left(t-\Delta t\right)+u\left(t\right)+m\left(t-\Delta t\right)$$
    (17) $${\hat{P}}^{-}\left(t\right)=\;{\hat{P}}^{+}\left(t-\Delta t\right)+M\left(t-\Delta t\right)$$
  (b) Estimation of noise statistics for the measurement system:
    (18) $$\hat{TT}\left(t\right)=\;H\;\left(t\right)\;\times \;{\hat{N}}^{-}\left(t\right)$$
    (19) $$r=\frac{1}{n}\sum_{t=1}^{n}\;\left[TT\left(t\right)-\hat{TT}\left(t\right)\right]$$
    (20) $$R=\frac{1}{n-1}\sum_{t=1}^{n}\;\left[\left(r(t)-r\right).{\left(r(t)-r\right)}^{T}-\left(\frac{n-1}{n}\right)H\left(t\right){\hat{P}}^{-}\left(t\right)H^{T}\left(t\right)\right]$$
    where *r* and *R* are the mean and covariance of the measurement noise, respectively, and *n* is the number of state noise samples.
  (c) Correction:
    (21) $$G\left(t\right)=\;{\hat{P}}^{-}\left(t\right)H{\left(t\right)}^{T}\;{\left[H\left(t\right){\hat{P}}^{-}\left(t\right)\;H{\left(t\right)}^{T}+R\left(t\right)\right]}^{-1}$$
  (d) Posterior state estimates:
    (22) $${\hat{N}}^{+}\left(t\right)={\hat{N}}^{-}\left(t\right)+G\left(t\right)\;\left[TT\left(t\right)-\hat{TT}\left(t\right)-r\left(t\right)\right]$$
    (23) $${\hat{P}}^{+}\left(t\right)={\hat{P}}^{-}\left(t\right)\times \left[1-H\;\left(t\right)\;G\;\left(t\right)\right]$$
  (e) Estimation of noise statistics for the state system:
    (24) $$m=\frac{1}{n}\sum_{t=1}^{n}\;\left[{\hat{N}}^{+}\left(t\right)-{\hat{N}}^{+}\left(t-\Delta t\right)-u\left(t\right)+m\left(t-\Delta t\right)\right]$$
    (25) $$M=\frac{1}{n-1}\sum_{t=1}^{n}\;\left[\left(m(t)-m\right).{\left(m(t)-m\right)}^{T}-\left(\frac{n-1}{n}\right){\hat{P}}^{+}\left(t-\Delta t\right)-{\hat{P}}^{+}\left(t\right)\right]$$
    where *m* and *M* are the mean and covariance of the state noise, respectively.
  (f) Next time step (t+Δt): When 5 new CVs traverse the link, return to step 2a.

## 3. Results and Discussion

This section evaluates and compares the three estimation approaches. The simulated data were generated for a signalized link under an oversaturation condition in which the traffic demand exceeds the link capacity. The free-flow speed is 40 km/h; the saturation flow rate is 1800 veh/h/lane, resulting in a traffic capacity of 855 veh/h given the cycle length and traffic signal’s green times; the speed-at-capacity is 32 km/h; and the jam density is 160 veh/km/lane. The traffic signal is operated at a cycle length of 120 s and a phase split of 50:50. The amber and all-red intervals are 3 s. To test the accuracy of the estimation approaches, the INTEGRATION microscopic traffic assignment and simulation software was used [43,44]. The relative root mean square error (RRMSE), presented in Equation (26), was used to evaluate the proposed estimation approaches.

(26) $$\mathrm{RRMSE}\left(\%\right)\;=\;100\;\frac{\sqrt{S\sum_{s=1}^{S}[{\hat{N}}^{+}\left(s\right)-N\left(s\right){]}^{2}}}{\sum_{s=1}^{S}N\left(S\right)}$$

where ${\hat{N}}^{+}\left(s\right)$ represents the estimated count of vehicles, N(s) represents the actual count of vehicles, and *S* is the overall number of estimations.

### 3.1. Performance of Estimation Approaches

The simulations were conducted with the same predefined initial conditions to obtain a fair comparison. The initial conditions are described in Table 1. It should be noted that each estimator requires specific initial variables. For instance, ${\hat{N}}^{+}\left(0\right)$, *R*, and ${\hat{P}}^{+}\left(0\right)$ are required for the KF approach. For all estimation approaches, the first estimate begins with an erroneous initial estimate of vehicle count (${\hat{N}}^{+}\left(0\right)$ = 5 veh), whereas the actual vehicle count is zero [4,7].

The three estimation approaches were evaluated using different CV LMPs, including 1%, 3%, 5%, 8%, 10%, 15%, 20%, 30%, 40%, 50%, 60%, 70%, 80% and 90%. For each LMP scenario, 100 random samples from the full data set were created using a Monte Carlo simulation. Table 2 presents the RRMSE values of the KF, AKF, and the PF approaches. The table indicates that estimation errors decrease with increasing LMP for all estimation approaches. The table also demonstrates that the KF outperforms the AKF and the PF approaches. For instance, for the scenario of 1% LMP, the vehicle count estimates were off by 30%, 48% and 64% using KF, AKF and PF, respectively.

The PF approach produces high RRMSE values at low LMPs (LMP <40%), while for the high-LMP scenarios, the PF produces RRMSE values close to the values obtained from the KF. Moreover, the AKF approach produces high errors, especially at very low LMPs (LMP <10%) and high LMPs (LMP >=70%). This demonstrates that the real-time estimates of the statistical noise values obtained from the AKF are not needed for the high-LMP scenarios, and the user may proceed with predefined statistical values due to low errors in the vehicle count estimates (low error in the ρ value). It was found that the high RRMSE error values produced from the AKF and PF approaches are mainly caused from assigning an inappropriate initial vehicle count estimate, as discussed in the next section.

**Figure 2:**
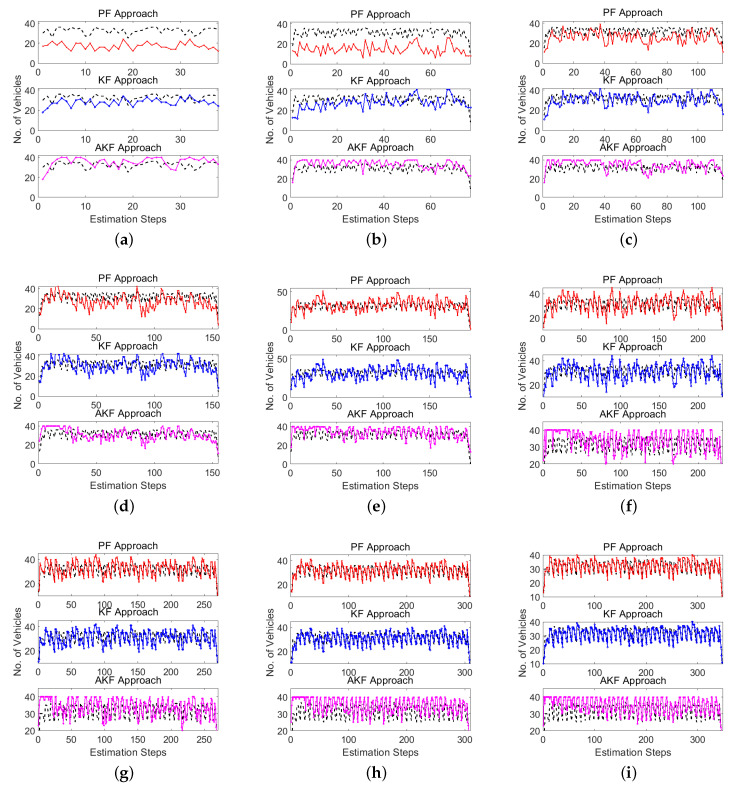
Actual and estimated vehicle counts at different LMP scenarios: (**a**) 10%, (**b**) 20%, (**c**) 30%, (**d**) 40%, (**e**) 50%, (**f**) 60%, (**g**) 70%, (**h**) 80% and (**i**) 90%.

Figure 2 presents the KF, AKF, and PF estimation outcomes with regard to the actual values at different LMPs (i.e., 10% to 90% with an increase of 10%). In each subfigure, three plots are generated to display the estimation approaches’ outcomes with regard to the actual values; the top one displays the PF outcomes, the middle one presents the KF outcomes, and the bottom one displays the AKF outcomes. The actual curve is represented by the dotted curve. In conclusion, the KF approach is recommended, as it produces the most accurate estimates in addition to its simplicity and applicability in the field. The next section will discuss the impact of the initial conditions on the performance of the various estimation approaches.

### 3.2. Impact of Initial Conditions

**Figure 3:**
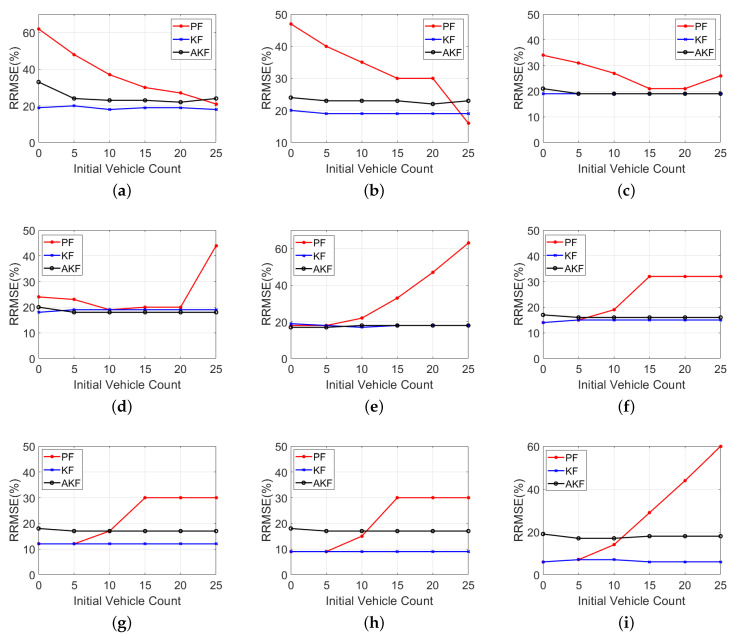
RRMSE values using various initial vehicle count estimates at different LMP scenarios: (**a**) 10%, (**b**) 20%, (**c**) 30%, (**d**) 40%, (**e**) 50%, (**f**) 60%, (**g**) 70%, (**h**) 80% and (**i**) 90%.

This section examines the effect of the choice of the initial conditions on the performance of the estimators, such as the initial vehicle count estimate ${\hat{N}}^{+}\left(0\right)$ and the *k* number of particles in the PF approach. First, different ${\hat{N}}^{+}\left(0\right)$ values were tested, from 0 to 25 at increments of 5, at different LMP scenarios, as presented in Table 3 and Table 4. Table 3 presents the RRMSE values when the ${\hat{N}}^{+}\left(0\right)$ is set to equal 0, 5, and 10 vehicles. Table 4 displays the RRMSE for the ${\hat{N}}^{+}\left(0\right)$ values of 15, 20, and 25 vehicles. The tables demonstrate that the RRMSE values are sensitive to the changes of the ${\hat{N}}^{+}\left(0\right)$ values. The tables also show that the PF is the most sensitive estimator to ${\hat{N}}^{+}\left(0\right)$ for all LMP scenarios. For instance, for the scenario of 1% LMP, the RRMSE is 81% when the simulation starts with 0 veh, while the RRMSE is 17% when ${\hat{N}}^{+}\left(0\right)$ is equal to 25. Therefore, starting the simulations with an appropriate initial estimate close to the truth value significantly improves the estimation accuracy since this helps the PF to quickly converge. In addition, the AKF seems to be sensitive to the ${\hat{N}}^{+}\left(0\right)$ with low LMP scenarios (LMP <=10%), while the choice of ${\hat{N}}^{+}\left(0\right)$ has a slight effect on the estimation accuracy for the scenarios with medium and high LMPs. For instance, for the scenario of 1% LMP, the RRMSE is 71% when the simulation starts with 0 veh, while the RRMSE is 21% when ${\hat{N}}^{+}\left(0\right)$ is equal to 25. Lastly, the tables show that the KF is the least-sensitive estimator to the ${\hat{N}}^{+}\left(0\right)$ value. Figure 3 summarizes the RRMSE values for nine LMP scenarios presented in Table 3 and Table 4.

This study also examined the choice of the number of particles, *k*, on the PF performance (i.e., k=10, 100, 200, 1000, and 2000), as presented in Table 5. The findings show that the estimation accuracy increases as the number of particles increases, especially at low LMPs. However, increasing the number of particles is associated with additional computational time. The PF is implemented in MATLAB R2019a on a Dell PC with 8.0 GB RAM. The computation time ranges between 0.2 and 1.6 s, with 10 particles for various LMPs; 1.1 and 3.0 s with 100 particles; 1.3 and 6.8 sec with 200 particles; 1.3 and 73 s with 1000 particles; 4 and 256 s with 2000 particles. The results in Table 5 show that the use of 1000 and 2000 particles slightly reduces the RRMSE values compared to the use of 200 particles; however, this comes at a very high computational cost. Therefore, the use of 200 particles is recommended in the PF approach.

## 4. Summary and Conclusions

The paper developed a nonlinear PF estimation approach to estimate the number of vehicles approaching a traffic signal based solely on CV data, with the aim of improving the estimation accuracy of linear state-of-the-art estimation approaches. This study introduced two linear approaches, KF and AKF, as benchmarks, to be compared with the proposed nonlinear PF approach. The results show that the KF produces the least error and accurately estimates the vehicle counts compared with the AKF and PF approaches. Consequently, to address the research problem appropriately, it is recommended to deploy the linear KF approach rather than the more complex AKF and PF approaches because of its simplicity and high-performance accuracy. In addition, the study investigated the sensitivity of the developed approaches to different factors, including the LMP of CVs, the initial vehicle count estimates, and the number of particles used in the PF approach. The results indicate that the estimation errors decrease as the LMP increases. Furthermore, the paper investigated the effect of the choice of the number of particles on the performance of the PF and showed that the PF estimation accuracy increases as the number of particles increases. However, this comes at the expense of significantly longer computational times. This can significantly impact the performance of the PF, requiring longer time to converge. The results demonstrate that the KF approach is the least sensitive to the initial vehicle count estimate, while the PF approach is the most sensitive to the initial vehicle count estimate and thus is the most suitable for the proposed application. Proposed future work entails integrating the KF approach with an adaptive traffic signal controller to quantify the impact of inaccuracies of the traffic stream density on the traffic signal controller performance.

## Figures and Tables

**Table 1:** Initial conditions for the Kalman filter (KF), adaptive KF (AKF), and particle filter (PF) approaches.

Initial Conditions	KF	AKF	PF
${\hat{N}}^{+}\left(0\right)$ (veh)	5	5	5
*R* (s${}^{2}$)	20	–	20
*V* (veh${}^{2}$)	–	–	5
*k* (# of part.)	–	–	200
${\hat{P}}^{+}\left(0\right)$ (veh${}^{2}$)	5	5	–
*m* (veh)	–	5	–

**Table 2:** Relative root mean square error (RRMSE) of KF, AKF, and PF approaches for different levels of market penetration rate (LMPs).

LMPs %	RRMSE (%)		
	KF	AKF	PF
1	30	48	64
3	25	34	60
5	23	32	56
8	23	28	52
10	19	24	48
15	19	24	42
20	18	23	40
30	18	19	30
40	18	18	22
50	18	17	18
60	14	16	15
70	12	17	12
80	9	17	9
90	6	17	7

**Table 3:** RRMSE values for the KF, AKF, and PF approaches using different initial vehicle count estimates (i.e., 0, 5 and 10) for different LMPs.

LMPs %	${\hat{N}}^{+}\left(0\right)$ = 0			${\hat{N}}^{+}\left(0\right)$ = 5			${\hat{N}}^{+}\left(0\right)$ = 10		
	KF	AKF	PF	KF	AKF	PF	KF	AKF	PF
1	34	71	81	30	48	64	27	36	51
3	28	49	78	25	34	60	23	26	47
5	26	45	73	23	32	56	23	27	44
8	24	33	69	23	28	52	23	27	41
10	19	33	62	19	24	48	20	24	37
15	21	29	55	19	24	42	20	23	37
20	20	24	47	18	23	40	19	23	35
30	19	21	34	18	19	30	19	19	27
40	18	20	24	18	18	22	19	18	19
50	19	17	18	18	17	18	17	17	22
60	14	17	14	14	16	15	15	16	19
70	12	18	12	12	17	12	12	17	17
80	9	18	9	9	17	9	9	17	15
90	6	17	6	6	17	7	7	17	14

**Table 4:** RRMSE values for the KF, AKF, and PF approaches using different initial vehicle count estimates (i.e., 15, 20 and 25) for different LMPs.

LMPs %	${\hat{N}}^{+}\left(0\right)$ = 15			${\hat{N}}^{+}\left(0\right)$ = 20			${\hat{N}}^{+}\left(0\right)$ = 25		
	KF	AKF	PF	KF	AKF	PF	KF	AKF	PF
1	23	32	36	20	23	24	19	21	17
3	22	26	33	20	25	24	20	24	19
5	21	25	31	20	23	26	19	24	20
8	21	27	33	22	26	26	21	26	23
10	20	24	30	20	24	27	19	26	22
15	19	23	30	19	24	26	19	23	18
20	19	23	30	19	23	30	19	23	16
30	19	19	21	19	19	21	19	19	26
40	19	18	20	19	18	20	19	18	44
50	18	17	33	18	17	47	18	17	33
60	15	16	32	15	16	32	15	16	32
70	12	17	30	12	17	30	12	17	30
80	9	17	30	9	17	30	9	17	30
90	7	17	29	7	17	44	7	17	29

**Table 5:** RRMSE values using different number of particles in the PF for different LMPs.

LMPs %	RRMSE (%)				
	*k* = 10	*k* = 100	*k* = 200	*k* = 1000	*k* = 2000
1	72	66	64	61	59
3	69	62	60	57	56
5	66	59	56	53	52
8	60	54	52	48	47
10	56	50	48	46	44
15	48	44	42	40	40
20	44	41	40	38	36
30	34	30	30	30	30
40	22	22	22	22	22
50	19	18	18	18	17
60	16	15	15	14	14
70	13	12	12	12	11
80	11	9	9	9	9
90	9	7	7	6	6
